# Unhealthy lifestyles and clusters status among 3637 adolescents aged 11–23 years: a school-based cross-sectional study in China

**DOI:** 10.1186/s12889-023-16197-3

**Published:** 2023-07-03

**Authors:** Yalin Song, Jingru Liu, Yize Zhao, Lu Gong, Qiuyuan Chen, Xili Jiang, Jiangtao Zhang, Yudan Hao, Huijun Zhou, Xiaomin Lou, Xian Wang

**Affiliations:** College of Public Health, Zhengzhou University, No.100 Science Avenue, Zhengzhou, 450001 Henan People’s Republic of China

**Keywords:** Life style, Adolescent, Cluster

## Abstract

**Background:**

Unhealthy lifestyles are risk factors for non-communicable diseases (NCDs) and tend to be clustered, with a trajectory that extends from adolescence to adulthood. This study investigated the association of diets, tobacco, alcohol, physical activity (PA), screen time (ST) and sleep duration (SD) in a total of six lifestyles, separately and as cumulative lifestyle scores, with sociodemographic characteristics among school-aged adolescents in the Chinese city of Zhengzhou.

**Methods:**

In the aggregate, 3,637 adolescents aged 11–23 years were included in the study. The questionnaire collected data on socio-demographic characteristics and lifestyles. Healthy and unhealthy lifestyles were identified and scored, depending on the individual score (0 and 1 for healthy and unhealthy lifestyles respectively), with a total score between 0 and 6. Based on the sum of the dichotomous scores, the number of unhealthy lifestyles was calculated and divided into three clusters (0–1, 2–3, 4–6). Chi-square test was used to analyze the group difference of lifestyles and demographic characteristics, and multivariate logistic regression was used to explore the associations between demographic characteristics and the clustering status of unhealthy lifestyles.

**Results:**

Among all participants, the prevalence of unhealthy lifestyles was: 86.4% for diet, 14.5% for alcohol, 6.0% for tobacco, 72.2% for PA, 42.3% for ST and 63.9% for SD. Students who were in university, female, lived in country (OR = 1.725, 95% CI: 1.241–2.398), had low number of close friends (1–2: OR = 2.110, 95% CI: 1.428–3.117; 3–5: OR = 1.601, 95% CI: 1.168–2.195), and had moderate family income (OR = 1.771, 95% CI: 1.208–2.596) were more likely to develop unhealthy lifestyles. In total, unhealthy lifestyles remain highly prevalent among Chinese adolescents.

**Conclusion:**

In the future, the establishment of an effective public health policy may improve the lifestyle profile of adolescents. Based on the lifestyle characteristics of different populations reported in our findings, lifestyle optimization can be more efficiently integrated into the daily lives of adolescents. Moreover, it is essential to conduct well-designed prospective studies on adolescents.

**Supplementary Information:**

The online version contains supplementary material available at 10.1186/s12889-023-16197-3.

## Introduction

Unhealthy lifestyles are becoming increasingly prevalent globally. Not only does it cause psychological health problems such as anxiety, depression and schizophrenia [1, 2], but it also leads to the development of non-communicable diseases (NCDs) such as type II diabetes, chronic respiratory diseases and cardiovascular diseases [3–5], which can cause nearly 41 million deaths each year, accounting for approximately 74% of global deaths. It has been shown that, the majority of the global NCDs burden (40%) was associated with modifiable behavioral risk factors, so reducing the risk factors associated with these diseases is an important way to control NCDs [6].

Adolescence is a crucial period not only for physical and physiological [7] development, but also for the establishment of lifestyle patterns [8, 9], which may persist into adulthood and impact long-term health outcomes [10]. However, studies have shown that the health status of adolescents in both developed and developing countries is not encouraging.

On the one hand, the prevalence of overweight and obesity among adolescents is increasing year by year [11], contributing to the development of cardiovascular and psychological diseases [12, 13]. These conditions are linked to unhealthy dietary habits including excessive consumption of sugar-sweetened beverages (SSBs) and fast food, as well as insufficient physical activity (PA) and excessive screen time (ST) [13]. Firstly, in terms of diet, nearly half of the 3525 school students in Greece had poor dietary habits, with the majority not eating fruit (69.3%) and vegetables (66.3%) on a daily basis, as well as consuming a high percentage of SSBs and sweets [14]. In China, 57.6% of adolescents aged 11–18 had not drunk milk or soy milk in past seven days and 20.3% drank SSBs at least once a day. In addition, fast food and fried food were popular among adolescents [15]. Secondly, WHO recommends at least 60 min (1 h) of moderate to vigorous physical activity (MVPA) per day for children and adolescents. However, the MVPA time for Chinese and American adolescents was 37.66 min/d [16] and 48 min/d [17], respectively, which were far below the recommended amount [18]. A significant association between more ST and lower PA levels has been reported among adolescents [19], suggesting that sedentary ST may occupy the time of other healthy activities. Currently, adolescents spend too much time on screens, including television, smartphones and computers. Adolescents in the United States use screens for an average of nine hours per day [20], and 85.8% of respondents in China National Nutrition and Health Survey (CNNHS) engaged in sedentary behavior for more than two hours per day [21].

On the other hand, regarding sleep duration, according to a report in 2020 on adolescent sleep duration published by the Institute of Psychology of the Chinese Academy of Sciences, 95.5% of primary school students slept an average of 8.7 h/d, 90.8% of middle school students slept 7.6 h/d and 84.1% of high school students slept 7.2 h/d. More than 80% of students failed to achieve the “national standard” for sleep duration [22]. In Mexican cities, adolescents aged 11–16 slept an average of 459.42 min (about 7.6 h) at night, and they often felt sleepy during the school day. Sleep deprivation was also very common among adolescents living in neighboring rural areas of Mexico [23]. Additionally, the behavior of alcohol and tobacco present among adolescents was relevant to low academic performance, involvement with other drugs, and aggressiveness [24].

Notably, it has been shown that clusters of unhealthy lifestyles are very common, and the synergistic impact of multiple unhealthy lifestyles can exceed the sum of their individual effects on health outcomes [25]. Several studies have explored the prevalence and risk factors for multiple lifestyles and clusters. For example, a Malaysian study reported that clusters of smoking, drinking, sedentary behavior (SB) and inadequate fruit and vegetable intake were more pronounced in boys. In the same study, adolescents who were Malay and had at least 3 close friends were less likely to exhibit health risk behaviors (HRBs) [26]. From a school-based cross-sectional study of Chinese adolescents aged 15–19, 63.7% of students aged 16–17 had a sub-healthy lifestyle (poor eating habits, sleep deprivation) and it was more common among boys than girls (54.6% vs 45.4%) [27]. In a systematic review conducted during 2014, eight studies revealed that females and low socioeconomic status (SES) were significant predictors for clusters of PA deficiency, SB and adverse dietary habits [28]. Therefore, a comprehensive understanding of the clusters of unhealthy lifestyles in adolescents is essential to improve their health status and reduce the risk of diseases in adulthood.

Over the past few decades, China has undergone profound economic, cultural and environmental transformations, and Chinese lifestyles have changed dramatically as well [29]. To name a few examples, consumption of foods high in sugar and fat has increased, and physical activity levels and sleep duration have declined along with increased screen time [30]. In short, the risk of high prevalence of unhealthy lifestyles persists. Adolescents grow up with multiple influences from family, school and society, which are important contributors to lifestyle [15]. However, as far as we know, research on unhealthy lifestyles in adolescents has been primarily focused on the association between certain lifestyles and cardiovascular disease, type II diabetes and other metabolic diseases [31, 32], lacking a comprehensive depiction of the current landscape of diverse lifestyles patterns and their clusters among adolescents. In addition, the objective factors that contribute to the occurrence of unhealthy lifestyles have not been adequately explored.

Given this context, the primary objective of this study was to assess the prevailing state of adolescents’ lifestyles and their clusters and to explore the risk factors and facilitators related to the lifestyles, in order to provide valuable insights for the development of effective interventions and strategies.

## Materials and methods

### Study design and participants

This study was conducted from September to December 2021 and was approved by Zhengzhou University Life Science Ethics Committee (protocol code ZZUIRB 2021–94). Informed consent was obtained from a parent and/or legal guardian and adolescent. This study was conducted in accordance with the Declaration of Helsinki. All methods were performed in accordance with the relevant guidelines and regulations.

Firstly, we selected a middle school, a vocational high school and a high school in the city and country areas of Zhengzhou, in addition to a university in the city. We then chose three grades in each of these middle schools, vocational high schools and high schools, and four grades in the university. Then, classes were randomly selected in whole clusters within grades, with 200 students selected from each grade, and if there were shortages, students in the remaining classes would be selected from until reaching 200 students.

After head teachers assisted in excluding students with serious organic and psychological disorders, trained researchers organized the completion of the questionnaires by students to ensure maximum completeness and authenticity. A total of 4351 questionnaires were completed. After excluding 714 questionnaires with missing or ineligible information, 3637 valid questionnaires were included in the analysis.

### Assessment of unhealthy lifestyles aggregations

The unhealthy lifestyles currently studied included six categories (unhealthy diet, lack of physical activity, smoking, drinking, excessive screen time and sleep deprivation). Based on previous reports [33, 34], scientific guidelines for different behaviors and practical assessments, a dichotomous variable was created for each behavioral question, with an answer of ‘0’ or ‘1’.

In terms of the definition of the clusters of risky behaviors, for each field of risky behavior, non-risky behaviors were assigned ‘0’ and risky behaviors were assigned ‘1’. There were six types of risky behaviors, and participants received one point for each of the six types of risky behaviors. Participants were divided into three categories: 0–1 unhealthy lifestyles clusters, 2–3 unhealthy lifestyles clusters and 4–6 unhealthy lifestyles clusters.

### Measurement of lifestyle behaviors

#### Dietary behaviors

Based on the results of the Mediterranean Diet Quality Index [35] and other domestic and international dietary health studies in the past five years, the Chinese Nutrition Guidelines have formulated guidelines for a rational diet for adolescents (10–24 years old) [36, 37]. Ate fast food at least once a week (1 point), not ate breakfast every day for the last week (1 point), ate less than two servings of vegetables per day for the last week (one serving = fist-size) (1 point), ate less than three servings of fruit per day for the last week (1 fruit = 1 medium apple, orange, etc.) (1 point), ate less than three servings of dried fruit per day for the last week (1 portion = palm size) (1 point), drank a sweet sugary beverage at least once a week in the last month (1 point).

We scored each dietary behavior, again created a dichotomous variable [38] with ‘0’ for each healthy dietary behavior and ‘1’ for each unhealthy dietary behavior. Lower scores reflected better dietary quality. In terms of unhealthy lifestyles clusters, we calculated the median dietary score of participants as 4 and defined healthy diet with a score below 4, and unhealthy diet with a score of 4 and above [39].

#### Tobacco and alcohol behaviors

Regarding tobacco behaviors, we defined the following three tobacco risk behaviors based on the nicotine dependence scale [40, 41] and passive smoking definition criteria [42], which are both suitable for multi-ethnic people of all ages: smoked at least one day in the last month, smoked at least one cigarette a day for the last month and exposed to smoke exhaled by smokers for at least 15 min at least one day a week for the last month. Of these, smoking at least one day in the last month was considered unhealthy tobacco behavior.

Regarding alcohol behaviors, according to the drinking scale [43] and the definition of binge drinking [44], we defined three types of alcohol behaviors: drank at least one drink in the last month, binge drank at least one day in the last month (binge drinking was defined as 4 or more drinks per day for women and 5 or more drinks per day for men), spit wine in the last month. Consistent with previous studies, drinking at least one drink in the last month was considered unhealthy alcohol behavior, based on the recommendation of nutrition guideline for Chinese.

#### Physical activity behaviors

We used the physical activity level scale (PARS-3) [45, 46] revised by Liang [47] for secondary school students (both middle and high school) and university students, which investigated the amount of exercise in three aspects: the intensity, time, frequency of physical activity participation. Exercise = intensity × time × frequency. Intensity and frequency were graded 1–5, and time was graded 0–4. The highest score was 100, and the lowest score was 0. Higher scores reflected better PA levels. The final scores were divided into four intervals (0, 1–19, 20–42, ≥ 43), and scores of 20 and above were defined as healthy PA behavior and scores below 20 were defined as unhealthy PA behavior.

#### Screen time

The screen time was measured by two questions [48]. One question was “How much time did you spend watching videos or playing computer games or doing something unrelated to study on weekdays in the last month?” The other question was “How much time did you spend watching videos or playing computer games or doing something unrelated to studying on weekends in the last month?” Each question has six choices, as follows: almost none, less than 1 h a day, less than 2 h a day, less than 3 h a day, less than 4 h a day, more than 4 h. Mean daily ST was calculated as [(weekdays ST × 5 + weekends ST × 2)/ 7]. According to the standard of American Academy of Pediatrics and previous studies [49], ST > 2 h/day was defined as excessive ST for children and adolescents over 6 years old.

#### Sleep duration

In terms of sleep duration, participants were asked to report the time they went to bed at night, the time they spent falling asleep, and the time they woke up in the morning on weekdays and weekends. Students were most likely to accumulate sleep debt on weekdays and pay off the accumulated sleep debt on weekends [50]. Therefore, to adjust for over-sleep on weekends, the corrected algorithm was used. Mean daily night sleep duration was calculated as [(mean weekdays night sleep duration × 5 + mean weekends night sleep duration × 2)/ 7]. According to Chinese national health requirements [51], sleep deficit was defined as less than 9 h/day for middle school students, less than 8 h/day for high school and vocational high school students, and less than 7 h/day for university students.

### Measurement of sociodemographic factors

The following information was included in the study: gender, school type (grouped as middle school, high school, vocational school, or university); residence (country or city); family population (grouped as 1–3, 4–5 or 6–15); whether only child or not (yes or no); parental educational levels (grouped as primary school and below, middle school or senior high school and above); number of close friends (0, 1–2, 3–5 or 6); self-reported family income (grouped as low, moderate or high); self-reported study burden (grouped as light, moderate or heavy).

### Statistical analysis

Descriptive analysis included the number of cases (N) and the percentage of categorical variables (%), which were used to show the frequency and rate of the sample. For a total of 19 lifestyle behaviors (6 of diet, 2 of tobacco, 3 of alcohol, 4 of PA, 2 of ST and 2 of SD), we used Pearson's chi-square test to assess the frequency of occurrence by sociodemographic characteristics. The Spearman correlation analysis was used to explore the links between six lifestyle categories. The pattern of clusters of the three categories (0–1, 2–3, 4–6) of unhealthy lifestyle behaviors was also tested using the chi-square test to compare differences between groups. Furthermore, logistic regression analysis was performed to estimate the associations between unhealthy lifestyles clusters and sociodemographic characteristics. SPSS 21.0 software was used for statistical analysis, and 0.05 was considered as a statistically significant difference.

## Results

### Participant characteristics

A total of 3637 participants aged 11–23 years were included in this analysis. 51% were female, 58.9% lived in country, and 87.8% were non-only child. Regarding school type, 27.6% were middle school students, 28.3% were high school students, 23.2% were vocational high school students, and 20.9% were university students. In addition, nearly half of the students' parents were junior high school educated (45.6% and 40.9%). 71.5% and 60.1% thought their family income and study burden were moderate, respectively.

### Comparison of unhealthy lifestyle behaviors among different participants

#### Dietary behaviors

The frequency of most dietary behaviors varied among different groups of people. Among all the participants, university students ate fast food the most (29.6%, *p* < 0.001), and about four-fifths of vocational high school students drank SSBs at least once a week (80.5%, *p* < 0.05). Boys ate breakfast (57.3% vs 52.5%, *p* < 0.05) and vegetables (51.8% vs 40.6%, *p* < 0.001) more often than girls. The consumption frequency of vegetables (48.7% vs 44.3%), fruits (19.7% vs 12.0%) and dried fruits (5.5% vs 2.7%) of students in city area was higher than those in country area. Fast food was more popular among adolescents who were only child (16.7%) and whose parents were highly educated (15.5%). Students with more than 6 close friends, high family income and light study burden consumed breakfast (60.6%, 59.7%, and 59.7%), vegetables (51.7%, 53.4%, and 58.6%) and fruits (19.5%, 24.9%, and 22.0%) more frequently **(**Table 1**).**

**Table 1:** Dietary behaviors among the participants (*N* = 3637)

Variables	Eat Fast food ≥ 3 times/w		Eat Breakfast < 7d/w		Eat Vegetables < 2 times/d		Eat Fruit < 3 times/d		Eat Dried fruits < 3 times/d		Drink SSB ≥ 1 time/w	
	**Yes**	**No**	**Yes**	**No**	**Yes**	**No**	**Yes**	**No**	**Yes**	**No**	**Yes**	**No**
	**N (%)**		**N (%)**		**N (%)**		**N (%)**		**N (%)**		**N (%)**	
**School type**												
Middle school	108(10.8)	896(89.2)	476(47.4)	528(52.6)	447(44.5)	557(55.0)	772(76.9)	232(23.1)	941(93.7)	63(6.3)	747(74.4)	257(25.6)
High school	39(3.8)	989(96.2)	410(39.9)	618(60.1)	699(68.0)	329(32.0)	905(88.0)	123(12.0)	992(96.5)	36(3.5)	791(76.9)	237(23.1)
Vocational High school	91(10.8)	754(89.2)	310(36.7)	535(63.3)	463(54.8)	382(45.2)	754(89.2)	91(10.8)	819(96.9)	26(3.1)	680(80.5)	165(19.5)
University	225(29.6)	535(70.4)	445(58.6)	315(41.4)	351(46.2)	409(53.8)	654(86.1)	106(13.9)	745(98.0)	15(2.0)	576(75.8)	184(24.2)
χ^2^	275.146**		93.164**		136.218**		70.933**		24.881**		10.092*	
**Gender**												
Male	239(13.4)	1544(86.6)	761(42.7)	1022(57.3)	859(48.2)	924(51.8)	1528(85.7)	255(14.3)	1695(95.1)	88(4.9)	1308(73.4)	475(26.6)
Female	224(12.1)	1630(87.9)	880(47.5)	974(52.5)	1101(59.4)	753(40.6)	1557(84.0)	297(16.0)	1802(97.2)	52(2.8)	1486(80.2)	368(19.8)
χ^2^	1.431		8.401*		45.948**		2.083		11.149**		23.544**	
**Residence**												
Country	255(11.9)	1888(88.1)	955(44.6)	1188(55.4)	1194(55.7)	949(44.3)	1885(88.0)	258(12.0)	2085(97.3)	58(2.7)	1664(77.6)	479(22.4)
City	208(13.9)	1286(86.1)	686(45.9)	808(54.1)	766(51.3)	728(48.7)	1200(80.3)	294(19.7)	1412(94.5)	82(5.5)	1130(75.6)	364(24.4)
χ^2^	3.243		0.651		6.998*		39.907**		18.41**		2.002	
**Family population**												
1–3	71(15.8)	378(84.2)	209(46.5)	240(53.5)	226(50.3)	223(49.7)	369(82.2)	80(17.8)	429(95.5)	20(4.5)	331(73.7)	118(26.3)
4–5	311(13.4)	2008(86.6)	1037(44.7)	1282(55.3)	1243(53.6)	1076(46.4)	1959(84.5)	360(15.5)	2228(96.1)	91(3.9)	1793(77.3)	526(22.7)
6–15	81(9.3)	788(90.7)	395(45.5)	474(54.5)	491(56.5)	378(43.5)	757(87.1)	112(12.9)	840(96.7)	29(3.3)	670(77.1)	199(22.9)
χ^2^	13.899**		0.561		4.748		6.184*		1.095		2.785	
**Only child**												
Yes	74(16.7)	370(83.3)	209(47.1)	235(52.9)	224(50.5)	220(49.5)	364(82.0)	80(18.0)	418(94.1)	26(5.9)	330(74.3)	114(25.7)
No	389(12.2)	2804(87.8)	1432(44.8)	1761(55.2)	1736(54.4)	1457(45.6)	2721(85.2)	472(14.8)	3079(96.4)	114(3.6)	2464(77.2)	729(22.8)
χ^2^	7.054*		0.779		2.049		3.17		5.502*		1.771	
**Father`s educational level**												
Primary school and below	66(9.9)	602(90.1)	286(42.8)	382(57.2)	369(55.2)	299(44.8)	590(88.3)	78(11.7)	647(96.9)	21(3.1)	500(74.9)	168(25.1)
Middle school	194(11.7)	1464(88.3)	755(45.5)	903(54.5)	907(54.7)	751(45.3)	1412(85.2)	246(14.8)	1607(96.9)	51(3.1)	1294(78.0)	364(22.0)
Senior high school and above	203(15.5)	1108(84.5)	600(45.8)	711(54.2)	684(52.2)	627(47.8)	1083(82.6)	228(17.4)	1243(94.8)	68(5.2)	1000(76.3)	311(23.7)
χ^2^	15.416**		1.772		2.486		11.499*		9.915*		3.071	
**Mother`s educational level**												
Primary school and below	108(11.1)	864(88.9)	422(43.4)	550(56.6)	538(55.3)	434(44.7)	864(88.9)	108(11.1)	940(96.7)	32(3.3)	738(75.9)	234(24.1)
Middle school	162(10.9)	1325(89.1)	679(45.7)	808(54.3)	813(54.7)	674(45.3)	1262(84.9)	225(15.1)	1440(96.8)	47(3.2)	1165(78.3)	322(21.7)
Senior high school and above	193(16.4)	985(83.6)	540(45.8)	638(54.2)	609(51.7)	569(48.3)	959(81.4)	219(18.6)	1117(94.8)	61(5.2)	891(75.6)	287(24.4)
χ^2^	20.958**		1.564		3.48		23.148**		8.341*		3.307	
**Close friends**												
0	5(7.5)	62(92.5)	36(53.7)	31(46.3)	35(52.2)	32(47.8)	58(86.6)	9(13.4)	66(98.5)	1(1.5)	44(65.7)	23(34.3)
1–2	104(10.4)	892(89.6)	520(52.2)	476(47.8)	591(59.3)	405(40.7)	863(86.6)	133(13.4)	971(97.5)	25(2.5)	735(73.8)	261(26.2)
3–5	231(13.3)	1509(86.7)	756(43.4)	984(56.6)	931(53.5)	809(46.5)	1493(85.8)	247(14.2)	1694(97.4)	46(2.6)	1369(78.7)	371(21.3)
> 6	123(14.7)	711(85.3)	329(39.4)	505(60.6)	403(48.3)	431(51.7)	671(80.5)	163(19.5)	766(91.8)	68(8.2)	646(77.5)	188(22.5)
χ^2^	9.892*		35.017**		22.479**		16.39**		54.411**		13.359*	
**Self-reported family income**												
Low	89(13.2)	584(86.8)	333(49.5)	340(50.5)	366(54.4)	307(45.6)	595(88.4)	78(11.6)	656(97.5)	17(2.5)	496(73.7)	177(26.3)
Moderate	320(12.3)	2279(87.7)	1148(44.2)	1451(56.2)	1424(54.8)	1175(45.2)	2216(85.3)	383(14.7)	2502(96.3)	97(3.7)	2003(77.1)	596(22.9)
High	54(14.8)	311(85.2)	160(43.8)	205(59.7)	170(46.6)	195(53.4)	274(75.1)	91(24.9)	339(92.9)	26(7.1)	295(80.8)	70(19.2)
χ^2^	1.956		6.355*		8.773*		34.096**		13.851**		7.052*	
**Self-reported study burden**												
Light	29(15.6)	157(84.4)	75(40.3)	111(59.7)	77(41.4)	109(58.6)	145(78.0)	41(22.0)	173(93.0)	13(7.0)	135(72.6)	51(27.4)
Moderate	274(12.5)	1913(87.5)	948(43.3)	1239(56.7)	1164(53.2)	1023(46.8)	1864(85.2)	323(14.8)	2104(96.2)	83(3.8)	1692(77.4)	495(22.6)
Heavy	160(12.7)	1104(87.3)	618(48.9)	646(51.1)	719(56.9)	545(43.1)	1076(85.1)	188(14.9)	1220(96.5)	44(3.5)	967(76.5)	297(23.5)
χ^2^	1.457		11.769*		16.629**		7.184*		5.435		2.315	

Abbreviation: *SSB* Sugar-sweet beverage

^*^P < 0.05, **P < 0.001

#### Drinking and smoking behaviors

Among the five behaviors of drinking, binge drinking, spitting wine, smoking and passive smoking, the subgroups with the highest percentage were vocational high school students, male, students in country area and with light study burden (*p* < 0.001 for all). In addition, non-only child (6.3% vs 3.8%) smoked at a higher rate than only child. Students whose parents had lower education level spit wine (6.3% vs 3.7% and 6.0% vs 4.1%) and smoked (7.0% vs 4.5% and 7.6% vs 4.6%) more frequently. Students with more than 6 close friends and high family income had the highest ratios of drinking (19.8% and 18.1%), binge drinking (12.0% and 9.8%) (*p* < 0.001 for all) **(**Table 2**)**.

**Table 2:** Alcohol and Tobacco behaviors among the participants (*N* = 3637)

Variables	Drinking		Binge drinking		Spit wine		Smoking		Passive smoking	
	**Yes**	**No**	**Yes**	**No**	**Yes**	**No**	**Yes**	**No**	**Yes**	**No**
	**N (%)**		**N (%)**		**N (%)**		**N (%)**		**N (%)**	
**School type**										
Middle school	109(10.9)	895(89.1)	60(6.0)	944(94.0)	27(2.7)	977(97.3)	41(4.1)	963(95.9)	219(21.8)	785(78.2)
High school	134(13.0)	894(87.0)	50(4.9)	978(95.1)	27(2.6)	1001(97.4)	38(3.7)	990(96.3)	205(19.9)	823(80.1)
Vocational high school	182(21.5)	663(78.5)	116(13.7)	729(86.3)	95(11.2)	750(88.8)	120(14.2)	725(85.8)	248(29.3)	97(70.7)
University	102(13.4)	658(86.6)	47(6.2)	713(93.8)	28(3.7)	732(96.3)	20(2.6)	740(97.4)	86(11.3)	674(88.7)
χ^2^	47.036**		92.759**		97.916**		131.824**		79.952**	
**Gender**										
Male	384(21.5)	1399(78.5)	202(11.3)	1581(88.7)	150(8.4)	1633(91.6)	188(10.5)	1595(89.5)	433(24.3)	1350(75.7)
Female	143(7.7)	1711(92.3)	71(3.8)	1783(96.2)	27(1.5)	1827(98.5)	31(1.7)	1823(98.3)	325(17.5)	1529(82.5)
χ^2^	140.178**		73.632**		95.002**		126.424**		25.141**	
**Residence**										
Country	326(15.2)	1817(84.8)	181(8.4)	1962(91.6)	131(6.1)	2012(93.9)	161(7.5)	1982(92.5)	466(21.7)	1677(78.3)
City	201(13.5)	1293(86.5)	92(6.2)	1402(93.8)	46(3.1)	1448(96.9)	58(3.9)	1436(96.1)	292(19.5)	1202(80.5)
χ^2^	2.197		6.638*		17.502**		20.505**		2.583	
**Family population**										
1–3	72(16.0)	377(84.0)	37(8.2)	412(91.8)	21(4.7)	428(95.3)	9(4.2)	430(95.8)	97(21.6)	352(78.4)
4–5	334(14.4)	1985(85.6)	176(7.6)	143(92.4)	118(5.1)	2201(94.9)	150(6.5)	2169(93.5)	458(19.7)	1861(80.3)
6–15	121(13.9)	748(86.1)	60(6.9)	809(93.1)	38(4.4)	831(95.6)	50(5.8)	819(94.2)	203(23.4)	666(76.6)
χ^2^	1.105		0.825		0.739		3.47		5.175	
**Only child**										
Yes	65(14.6)	379(85.4)	30(6.8)	414(93.2)	19(4.3)	425(95.7)	17(3.8)	427(96.2)	93(20.9)	351(79.1)
No	462(14.5)	2731(85.5)	243(7.6)	2950(92.4)	158(4.9)	3035(95.1)	202(6.3)	2991(93.7)	665(20.8)	2528(79.2)
χ^2^	0.009		0.409		0.377		4.297*		0.003	
**Father`s educational level**										
Primary school and below	105(15.7)	563(84.3)	60(9.0)	608(91.0)	42(6.3)	626(93.7)	47(7.0)	621(93.0)	152(22.8)	516(77.2)
Middle school	242(14.6)	1416(85.4)	126(7.6)	1532(92.4)	87(5.2)	1571(94.8)	113(6.8)	1545(93.2)	358(21.6)	1300(78.4)
Senior high school and above	180(13.7)	1131(86.3)	87(6.6)	1224(93.4)	48(3.7)	1263(96.3)	59(4.5)	1252(95.5)	248(18.9)	1063(81.1)
χ^2^	1.44		3.546		7.545*		8.422*		4.992	
**Mother`s educational level**										
Primary school and below	152(15.6)	820(84.4)	80(8.2)	892(91.8)	58(6.0)	914(94.0)	74(7.6)	898(92.4)	214(22.0)	758(78.0)
Middle school	200(13.4)	1287(86.6)	101(6.8)	1386(93.2)	71(4.8)	1416(95.2)	91(6.1)	1396(93.9)	319(21.5)	1168(78.5)
Senior high school and above	175(14.9)	1003(85.1)	92(7.8)	1086(92.2)	48(4.1)	1130(95.9)	54(4.6)	1124(95.4)	225(19.1)	953(80.9)
χ^2^	2.459		1.983		4.165		8.678*		3.315	
**Close friends**										
0	11(16.4)	56(83.6)	7(10.4)	60(89.6)	7(10.4)	60(89.6)	6(9.0)	61(91.0)	23(34.3)	44(65.7)
1–2	120(12.0)	876(88.0)	53(5.3)	943(94.7)	41(4.1)	955(95.9)	32(3.2)	964(96.8)	216(21.7)	780(78.3)
3–5	231(13.3)	1509(86.7)	113(6.5)	1627(93.5)	68(3.9)	1675(96.1)	98(5.6)	1642(94.4)	344(19.8)	1396(80.2)
> 6	165(19.8)	669(80.2)	100(12.0)	734(88.0)	61(7.3)	773(92.7)	83(10.0)	751(90.0)	175(20.1)	659(79.0)
χ^2^	25.93**		34.405**		19.963**		38.138**		9.039*	
**Self-reported family income**										
Low	122(18.1)	551(81.9)	66(9.8)	607(90.2)	48(7.1)	625(92.9)	49(7.3)	624(92.7)	158(23.5)	515(76.5)
Moderate	345(13.3)	2254(86.7)	177(6.8)	2422(93.2)	113(4.3)	2486(95.7)	155(6.0)	2444(94.0)	533(20.5)	2066(79.5)
High	60(16.4)	305(83.6)	30(8.2)	335(91.8)	16(4.4)	349(95.6)	15(4.1)	350(95.9)	67(18.4)	298(81.6)
χ^2^	11.406*		7.211*		9.156*		4.259		4.375	
**Self-reported study burden**										
Light	51(27.4)	135(72.6)	31(16.7)	155(83.3)	22(11.8)	164(88.2)	31(16.7)	155(83.3)	59(31.7)	127(68.3)
Moderate	288(13.2)	1899(86.8)	147(6.7)	2040(93.3)	99(4.5)	2088(95.5)	127(5.8)	2060(94.2)	413(18.9)	1774(81.1)
Heavy	188(14.9)	1076(85.1)	95(7.5)	1169(92.5)	56(4.4)	1208(95.6)	61(4.8)	1203(95.2)	286(22.6)	978(77.4)
χ^2^	28.326**		24.421**		20.534**		40.617**		20.863**	

^*^P < 0.05, **P < 0.001

#### Physical activities

The subgroups that reported insufficient PA (scores of 19 and below) were: students who were in high school (73.2%), female (84.7%), lived in country (79.3%), non-only child (73.1%), with low literacy parents (79.5%, 79.5%), low family income (76.1%) and heavy study burden (72.1%) **(**Table 3**)**.

**Table 3:** Physical activities behaviors among the participants (*N* = 3637)

Variables	Physical Activities Score			
	**0**	**1–19**	**20–42**	** ≥ 43**
	**N (%)**	**N (%)**	**N (%)**	**N (%)**
**School type**				
Middle school	129(12.9)	520(51.8)	213(21.2)	142(14.1)
High school	139(13.6)	613(59.6)	213(20.7)	63(6.1)
Vocational high school	123(14.5)	495(58.6)	156(18.5)	71(8.4)
University	86(11.3)	522(68.7)	94(12.4)	58(7.6)
χ^2^	86.328**			
**Gender**				
Male	171(9.6)	885(49.6)	446(25.0)	281(15.8)
Female	306(16.5)	1265(68.2)	230(12.4)	53(2.9)
χ^2^	328.768**			
**Residence**				
Country	340(15.9)	1358(63.4)	320(14.9)	125(5.8)
City	137(9.2)	792(53.0)	356(23.8)	209(14.0)
χ^2^	147.319**			
**Family population**				
1–3	50(11.1)	270(60.1)	81(18.0)	48(10.8)
4–5	292(12.6)	1339(57.7)	463(20.0)	225(9.7)
6–15	135(15.5)	541(62.3)	132(15.2)	61(7.0)
χ^2^	22.036*			
**Only child**				
Yes	49(11.0)	246(55.4)	87(19.6)	62(14.0)
No	428(13.5)	1904(59.6)	589(18.4)	272(8.5)
χ^2^	15.706*			
**Father`s educational level**				
Primary school and below	115(17.2)	416(62.3)	89(13.3)	48(7.2)
Middle school	232(14.0)	990(59.7)	305(18.4)	131(7.9)
Senior high school and above	130(9.9)	744(56.8)	282(21.5)	155(11.8)
χ^2^	54.072**			
**Mother`s educational level**				
Primary school and below	151(15.5)	622(64.0)	128(13.2)	71(7.3)
Middle school	197(13.3)	864(58.1)	304(20.4)	122(8.2)
Senior high school and above	129(11.1)	664(56.3)	244(20.6)	141(12.0)
χ^2^	50.461**			
**Close friends**				
0	20(29.8)	29(43.3)	13(19.4)	5(7.5)
1–2	171(17.2)	599(60.1)	153(15.4)	73(7.3)
3–5	208(12.0)	1084(62.3)	310(17.8)	138(7.9)
> 6	78(9.4)	438(52.5)	200(24.0)	118(14.1)
χ^2^	98.235**			
**Self-reported family income**				
Low	102(15.2)	410(60.9)	108(16.0)	53(7.9)
Moderate	333(12.8)	1564(60.2)	478(18.4)	224(8.6)
High	42(11.5)	176(48.2)	90(24.7)	57(15.6)
χ^2^	39.466**			
**Self-reported study burden**				
Light	15(8.1)	94(50.5)	48(25.8)	29(15.6)
Moderate	288(13.2)	1319(60.3)	385(17.6)	195(8.9)
Heavy	174(13.8)	737(58.3)	243(19.2)	110(8.7)
χ^2^	22.448*			

^*^P < 0.05, **P < 0.001

#### Screen time and sleep duration

Regarding ST, the occurrence of ST exceeding two hours was more prevalent during weekends compared to weekdays across all demographic groups. The subgroups with the highest frequency of excessive ST on weekdays and weekends were: students who were in university (69.7% and 78.6%), lived in country (33.8% and 70.6%), with low literacy mothers (36.0% and 72.1%) and low family income (43.8% and 73.6%) (*p* < 0.001 for all). In addition, only child (38.1% vs 28.9%) and students in small families (38.3% vs 27.0%) had a higher frequency of ST during the weekdays compared to non-only child and students in large families (*p* < 0.001 for both), while there were no significant differences on weekends. Students with low literacy fathers (70.8% vs 64.7%, *p* < 0.05) had a higher frequency of ST on weekends compared to those with high literacy fathers, while there was no significant difference on weekdays. No differences were identified by gender and by number of close friends in terms of ST on weekdays and weekends.

Regarding SD, all categories of people had higher SD on weekends than on weekdays. The subgroups with the highest frequency of inadequate SD on weekdays and weekends respectively were the same: students who were in high school (99.0% and 53.9%, *p* < 0.001 for both), male (77.2%, *p* < 0.05 and 39.0%, *p* < 0.001), lived in the city (82.1%, *p* < 0.001 and 36.6%, *p* < 0.05), not only child (76.4%, *p* < 0.001 and 34.2%, *p* < 0.05), had high family income (86.8%, *p* < 0.001 and 37.8%, *p* < 0.05) and had heavy study burden (78.8% and 36.0%, *p* < 0.05 for both). Students with large families (80.4% vs 67.3%, *p* < 0.001) had a higher frequency of SD deficits on weekdays than those with small families, while there was no significant difference in SD on weekends. No differences in the frequency of SD deficits were identified in the number of close friends **(**Table 4**)**.

**Table 4:** Unhealthy screen time and sleep duration behaviors among the participants (*N* = 3637)

Variables	Weekday Screen Time ≥ 2 h/day		Weekend Screen Time ≥ 2 h/day		Not Enough Weekday Sleep Duration		Not Enough Weekend Sleep Duration	
	**Yes**	**No**	**Yes**	**No**	**Yes**	**No**	**Yes**	**No**
	**N (%)**		**N (%)**		**N (%)**		**N (%)**	
**School type**								
Middle school	194(19.3)	810(80.7)	572(57.0)	432(43.0)	972(96.8)	32(3.2)	416(41.4)	588(58.6)
High school	61(5.9)	967(94.1)	624(60.7)	404(39.3)	1018(99.0)	10(1.0)	554(53.9)	474(46.1)
Vocational high school	306(36.2)	539(63.8)	647(76.6)	198(23.4)	551(65.2)	294(34.8)	148(17.5)	697(82.5)
University	530(69.7)	230(30.3)	597(78.6)	163(21.4)	206(27.1)	554(72.9)	104(13.7)	656(86.3)
χ^2^	925.062**		145.163**		1566.102**		450.447**	
**Gender**								
Male	557(31.2)	1226(68.8)	1186(66.5)	597(33.5)	1376(77.2)	407(22.8)	696(39.0)	1087(61.0)
Female	534(28.8)	1320(71.2)	1254(67.6)	600(32.4)	1371(73.9)	483(26.1)	526(28.4)	1328(71.6)
χ^2^	2.57		0.517		5.115*		46.331**	
**Residence**								
Country	724(33.8)	1419(66.2)	1512(70.6)	31(29.4)	1521(71.0)	622(29.0)	675(31.5)	1468(68.5)
City	367(24.6)	1127(75.4)	928(62.1)	566(37.9)	1226(82.1)	268(17.9)	547(36.6)	947(63.4)
χ^2^	35.633**		28.401**		58.538**		10.324*	
**Family population**								
1–3	172(38.3)	277(1.7)	303(67.5)	146(32.5)	302(67.3)	147(32.7)	144(32.1)	305(67.9)
4–5	684(29.5)	1635(70.5)	1569(67.7)	750(32.3)	1746(75.3)	573(24.7)	772(33.3)	1547(66.7)
6–15	235(27.0)	634(73.0)	568(65.4)	301(34.6)	699(80.4)	170(19.6)	306(35.2)	563(64.8)
χ^2^	18.657**		1.545		28.007**		1.583	
**Only child**								
Yes	169(38.1)	275(61.9)	295(66.4)	149(33.6)	306(68.9)	138(31.1)	130(29.3)	314(70.7)
No	922(28.9)	2271(71.1)	2145(67.2)	1048(32.8)	2441(76.4)	752(23.6)	1092(34.2)	2101(65.8)
χ^2^	15.668**		0.096		11.957**		4.23*	
**Father`s educational level**								
Primary school and below	224(33.5)	444(66.5)	473(70.8)	195(29.2)	475(71.1)	193(28.9)	233(34.9)	435(65.1)
Middle school	478(28.8)	1180(71.2)	1119(67.5)	539(32.5)	1272(76.7)	386(23.3)	559(33.7)	1099(66.3)
Senior high school and above	89(29.7)	922(70.3)	848(64.7)	463(35.3)	1000(76.3)	311(23.7)	430(32.8)	881(67.2)
χ^2^	5.119		7.742*		8.733*		0.877	
**Mother`s educational level**								
Primary school and below	350(36.0)	622(64.0)	701(72.1)	271(27.9)	683(70.3)	289(29.7)	311(32.0)	661(68.0)
Middle school	402(27.0)	1085(73.0)	994(66.8)	493(33.2)	1186(79.8)	301(20.2)	527(35.4)	960(64.6)
Senior high school and above	339(28.8)	839(71.2)	745(63.2)	433(36.8)	878(74.5)	300(25.5)	384(32.6)	794(67.4)
χ^2^	23.776**		19.072**		29.579**		3.909**	
**Close friends**								
0	18(26.9)	49(73.1)	40(59.7)	27(40.3)	58(86.6)	9(13.4)	24(35.8)	43(64.2)
1–2	288(28.9)	708(71.1)	655(65.8)	341(34.2)	782(78.5)	214(21.5)	350(35.1)	646(64.9)
3–5	536(30.8)	1204(69.2)	1193(68.6)	547(31.4)	1273(73.2)	467(26.8)	581(33.4)	1159(66.6)
> 6	249(29.9)	585(70.1)	552(66.2)	282(33.8)	634(76.0)	200(24.0)	267(32.0)	567(68.0)
χ^2^	1.416		4.469		14.606		2.182	
**Self-reported family income**								
Low	295(43.8)	378(26.2)	495(73.6)	178(26.4)	423(62.9)	250(37.1)	195(29.0)	478(71.0)
Moderate	719(27.7)	1880(72.3)	1736(66.8)	863(33.2)	2007(77.2)	592(22.8)	889(34.2)	1710(65.8)
High	77(21.1)	288(78.9)	209(57.3)	156(42.7)	317(86.8)	48(13.2)	138(37.8)	227(62.2)
χ^2^	81.864**		28.8**		87.847**		9.778*	
**Self-reported study burden**								
Light	61(32.8)	125(67.2)	109(58.6)	77(41.4)	138(74.2)	48(25.8)	44(23.7)	142(76.3)
Moderate	681(31.1)	1506(68.9)	1468(57.1)	719(32.9)	1613(73.8)	574(26.2)	723(33.1)	1464(66.9)
Heavy	349(27.6)	915(72.4)	863(68.3)	401(31.7)	996(78.8)	268(21.2)	455(36.0)	809(64.0)
χ^2^	5.478		6.874*		11.213*		11.786*	

^*^P < 0.05, **P < 0.001

### Correlation between categories of lifestyles

As demonstrated in Figure S2 and Table S2, alcohol drinking was positively associated with tobacco use (*r* = 0.392, *p* < 0.05), unhealthy diet (*r* = 0.049, *p* < 0.01) and excessive ST (*r* = 0.104, *p* < 0.01). Unhealthy diet was positively related to inadequate PA (*r* = 0.086, *p* < 0.01) and excessive ST (*r* = 0.041, *p* < 0.05).

### The fractions of healthy and unhealthy lifestyles for different categories of participants

**Fig. 1:**
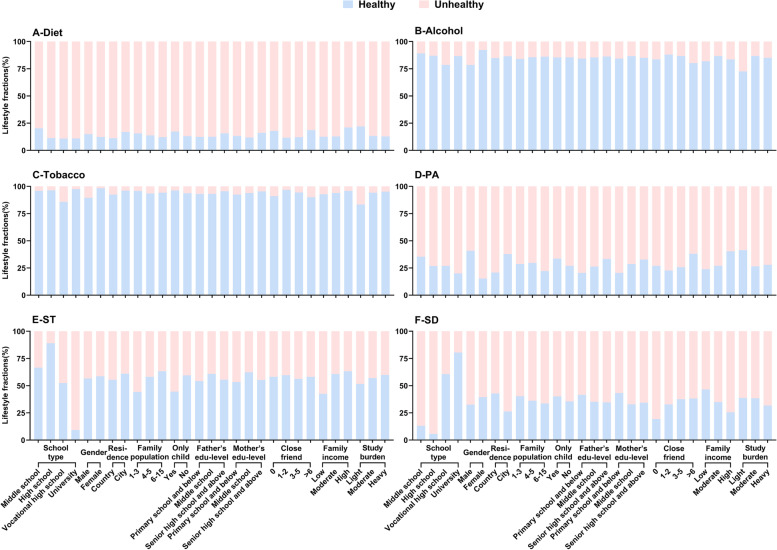
Six categories of lifestyles among participants (*N* = 3637). Figure 1 shows the proportion of healthy and unhealthy counts by sociodemographic characteristics in each lifestyle. Association of socio-demographic characteristics with proportion of dietary behavior (Fig. 1A), alcohol behavior (Fig. 1B), tobacco behavior (Fig. 1C), PA behavior (Fig. 1D), ST behavior (Fig. 1E) and SD behavior (Fig. 1F). Unhealthy dietary behavior: Diet quality score of 4 and above; Unhealthy alcohol behavior: Drank at least one glass of wine in the last month; Unhealthy tobacco behavior: Smoked at least one day in the last month; Unhealthy PA behavior: PARS-3 scores between 0 and 19; Unhealthy ST behavior: Daily average ST > 2 h in the last month; Unhealthy SD behavior: Sleep deprivation in the last week (less than 9 h/d for middle school students, less than 8 h/d for high school and vocational high school students, less than 7 h/d for university students). Abbreviations: PA, physical activity; PARS-3, physical activity rating scale-3; ST, screen time; SD, sleep duration

Figure 1 showed students in vocational high schools exhibited the highest prevalence of unhealthy diet, alcohol and tobacco behaviors, Conversely, university students displayed the highest rates of unhealthy PA and ST behaviors, while high school students had the highest rates of inadequate SD. Male students demonstrated higher rates of unhealthy alcohol, tobacco and SD behaviors compared to female students., but had healthier diet and PA behaviors than female students. No difference in ST between male and female were identified. Students resided in city exhibited less healthy ST behavior compared to those in country, but their diet, tobacco, PA, and SD behaviors were all healthier than those lived in country. No differences were identified between country and city in terms of alcohol behavior.

Students lived in large families had higher rates of unhealthy PA behavior and those lived in small families had higher rates of unhealthy ST behavior. Only child had higher rates of unhealthy ST behavior, but had healthier diet, tobacco and PA behaviors than non-only child. No differences in alcohol and SD behaviors were identified between only child and non-only child. Students with low parental literacy had the highest rates of unhealthy tobacco, PA, and ST behaviors, but their SD behavior were the healthiest. In addition, students with high parental literacy were the healthiest for diet behavior. No differences were identified in alcohol behavior.

Students with more than six close friends were the healthiest in terms of diet, PA and ST behaviors, while students with 1–2 close friends were the healthiest in terms of alcohol and tobacco behaviors. No difference was identified in ST behavior. Students with higher family income are healthier than those with lower family incomes in terms of diet, alcohol, PA, and ST behaviors, but had higher rates of SD deficits. No difference was identified in tobacco behavior. Students with heavy study burden had the highest rates of unhealthy diet, PA, SD, but students with light study burden had the highest rates of unhealthy alcohol and tobacco behaviors. No differences were found for study burden in ST behavior.

### Associations between socio-demographic variables and clustering of behavioral risk factors among participants

Table 5 shows the number of clusters of unhealthy lifestyles for different participants. The majority of people had 2–3 unhealthy lifestyles. The subgroups most likely to report 4–6 unhealthy lifestyles were: students were in vocational high school (26.2%), male (24.6%), lived in country (22.3%), with low literacy mothers (22.5%), with no close friend (29.8%), with low family income (25.4%) and heavy study burden (26.8%) (all *p* < 0.05). No significant differences between unhealthy lifestyles clustering and family population, only child, father’s educational level was identified.

**Table 5:** Pearson's chi-square test analysis of unhealthy lifestyles clustering among different participants

Variables	Number of unhealthy lifestyles clusters			χ^2^	*P*
	**0–1**	**2–3**	**4–6**		
	**N (%)**	**N (%)**	**N (%)**		
**School type**					
Middle school	99(9.9)	67(67.4)	228(22.7)	118.568	< 0.001**
High school	35(3.4)	843(82.0)	150(14.6)		
Vocational high school	80(9.5)	543(64.3)	222(26.2)		
University	23(3.0)	589(77.5)	148(19.5)		
**Gender**					
Male	142(8.0)	1202(67.4)	439(24.6)	53.74	< 0.001**
Female	95(5.1)	1450(78.2)	309(16.7)		
**Residence**					
Country	109(5.1)	1556(72.6)	478(22.3)	5.246	< 0.001**
City	128(8.6)	1096(73.4)	270(18.0)		
**Family population**					
1–3	30(6.7)	314(69.9)	105(23.4)	5.246	0.263
4–5	159(6.9)	1703(73.4)	457(19.7)		
6–15	48(5.5)	635(73.1)	186(21.4)		
**Only child**					
Yes	37(8.4)	314(70.7)	93(20.9)	2.932	0.231
No	200(6.3)	2338(73.2)	655(20.5)		
**Father`s educational level**					
Primary school and below	35(5.2)	474(71.0)	159(23.8)	8.018	0.091
Middle school	108(6.5)	1210(73.0)	340(20.5)		
Senior high school and above	94(7.2)	968(73.8)	49(19.0)		
**Mother`s educational level**					
Primary school and below	45(4.6)	709(72.9)	218(22.5)	10.005	0.04*
Middle school	104(7.0)	1092(73.4)	291(19.6)		
Senior high school and above	88(7.5)	851(72.2)	239(20.3)		
**Close friends**					
0	5(7.5)	42(62.7)	20(29.8)	32.107	< 0.001**
1–2	46(4.6)	753(75.6)	197(19.8)		
3–5	101(5.8)	1286(73.9)	353(20.3)		
> 6	85(10.2)	571(68.5)	178(21.3)		
**Self-reported family income**					
Low	37(5.5)	465(69.1)	171(25.4)	38.306	< 0.001**
Moderate	155(6.0)	1949(75.0)	495(19.0)		
High	45(12.3)	238(65.2)	82(22.5)		
**Self-reported study burden**					
Light	20(10.8)	116(62.4)	50(26.8)	15.158	0.004*
Moderate	149(6.9)	1604(73.3)	434(19.8)		
Heavy	68(5.4)	932(73.7)	264(20.9)		

^*^P < 0.05, **P < 0.001

Compared to university students, middle school students and vocational high school students were identified to be negatively associated with 2–3 unhealthy lifestyles clusters (OR = 0.307, 95% CI: 0.188–0.502 and OR = 0.227, 95% CI: 0.136–0.379, respectively) and 4–6 unhealthy lifestyles clusters (OR = 0.423, 95% CI: 0.250–0.717 and OR = 0.364, 95% CI: 0.211–0.627, respectively). Male students reported 0.702-fold (95% CI: 0.528–0.933) 2–3 unhealthy lifestyles clusters compared to female students. Lived in country increased the risk of 2–3 unhealthy lifestyles clusters (OR = 1.725, 95% CI: 1.241–2.398) and 4–6 unhealthy lifestyles clusters (OR = 2.043, 95% CI: 1.424–2.931). Compared to students with more than 6 close friends, students with 2–3 and 4–6 close friends reported respectively 2.110-fold (95% CI: 1.428–3.117) and 1.601-fold (95% CI: 1.168–2.195) 2–3 unhealthy lifestyles clusters, as well as reporting respectively 1.962-fold (95% CI: 1.282–3.003) and 1.543-fold (95% CI: 1.089–2.186) 4–6 unhealthy lifestyles clusters. At the same time, there was positive association between moderate family income and 2–3 unhealthy lifestyles clusters (OR = 1.771, 95% CI: 1.208–2.596) **(**Table 6**)**.

**Table 6:** Independent influential factors for unhealthy lifestyle clustering in the participants

Variables	2–3 unhealthy lifestyles clusters					4–6 unhealthy lifestyles clusters				
	***B***	***SE***	***Wald χ***^***2***^	***OR***	***95% CI***	***B***	***SE***	***Wald χ***^***2***^	***OR***	***95% CI***
**School type**										
Middle school	-1.181	0.251	22.149**	**0.307**	**0.188–0.502**	-0.860	0.268	10.272*	**0.423**	**0.250–0.716**
High school	0.020	0.283	0.005	1.020	0.586–1.776	-0.288	0.302	0.910	0.749	0.414–1.356
Vocational high school	-1.482	0.261	32.318**	**0.227**	**0.136–0.379**	-1.010	0.277	13.285**	**0.364**	**0.211–0.627**
University				1(ref)					1(ref)	
**Gender**										
Male	-0.354	0.145	5.948*	**0.702**	**0.528–0.933**	0.137	0.159	0.739	1.146	0.840–1.565
Female				1(ref)					1(ref)	
**Residence**										
Country	0.545	0.168	10.536*	**1.725**	**1.241–2.398**	0.715	0.184	15.053**	**2.043**	**1.424–2.931**
City				1(ref)					1(ref)	
**Family population**										
1–3	0.193	0.345	0.311	1.213	0.616–2.386	0.471	0.371	1.617	1.602	0.775–3.311
4–5	-0.108	0.178	0.366	0.898	0.633–1.273	-0.189	0.192	0.971	0.828	0.568–1.206
6–15				1(ref)					1(ref)	
**Only child**										
Yes	-0.429	0.296	2.097	0.651	0.364–1.164	-0.529	0.326	2.623	0.589	0.311–1.117
No				1(ref)					1(ref)	
**Father`s educational level**										
Primary school and below	-0.072	0.248	0.084	0.930	0.572–1.513	0.149	0.267	0.313	1.161	0.688–1.959
Middle school	-0.019	0.183	0.010	0.982	0.685–1.406	0.077	0.201	0.147	1.080	0.728–1.601
Senior high school and above				1(ref)					1(ref)	
**Mother`s educational level**										
Primary school and below	0.405	0.237	2.914	1.499	0.942–2.387	0.190	0.256	0.553	1.209	0.733–1.996
Middle school	0.072	0.189	0.146	1.075	0.742–1.557	-0.190	0.208	0.838	0.827	0.550–1.243
Senior high school and above				1(ref)					1(ref)	
**Close friends**										
0	0.351	0.502	0.490	1.421	0.532–3.798	0.697	0.530	1.732	2.008	0.711–5.669
1–2	0.747	0.199	14.057**	**2.110**	**1.428–3.117**	0.674	0.217	9.647*	**1.962**	**1.282–3.003**
3–5	0.471	0.161	8.556*	**1.601**	**1.168–2.195**	0.434	0.178	5.965*	**1.543**	**1.089–2.186**
> 6				1(ref)					1(ref)	
**Self-reported family income**										
Low	0.299	0.261	1.312	1.348	0.809–2.248	0.294	0.284	1.070	1.341	0.769–2.340
Moderate	0.572	0.195	8.581*	**1.771**	**1.208–2.596**	0.283	0.218	1.678	1.327	0.865–2.034
High				1(ref)					1(ref)	
**Self-reported study burden**										
Light	-0.062	0.289	0.046	0.940	0.533–1.657	-0.003	0.313	0	0.997	0.540–1.841
Moderate	0.001	0.160	0	1.001	0.731–1.371	-0.176	0.173	1.027	0.839	0.597–1.178
Heavy				1(ref)					1(ref)	

^*^P < 0.05, **P < 0.001

*OR* Odds ratio, *CI* Confidence interval

Relative risk ratio is estimated using logistic regression. Reference category: 0–1 risk factor

## Discussion

While the significance of unhealthy lifestyles in elevating the risk of NCDs has been extensively acknowledged [4, 5, 24], we know very little about the associations between lifestyles status and clusters and the socio-demographic characteristics. Therefore, this study had two main objectives. Firstly, we aimed to investigate the present status of lifestyle among diverse populations by categorizing them into six lifestyle domains (diet, tobacco, alcohol, PA, ST, SD), and to provide a comprehensive delineation of each category. Secondly, we also decided three cluster patterns of unhealthy lifestyles (0–1, 2–3, 4–6) and assessed the associated risk groups.

The results of the study showed a significant prevalence of unhealthy diet, insufficient PA, excessive SD and sleep deprivation among adolescents aged 11–23 years in China. In contrast, the prevalence of alcohol and tobacco behaviors was lower. Additionally, clusters of unhealthy lifestyles were very common, with around 90% of adolescents having two or more unhealthy lifestyles. Previous studies have shown similar phenomena, however, comparisons between different studies should be made with caution due to variations in the categories of unhealthy lifestyles investigated, the cut-off points for defining unhealthy lifestyles, and the target population recruited. Malaysian researchers used the same methods for defining smoking, alcohol drinking, and ST in a study of 3578 adolescents which showed a higher prevalence of alcohol use (14.5% vs 5.0%) and lower ratios of smoking and adverse ST (6.0% vs 14.6% and 42.2% vs 56.6%) than our study [25]. In terms of the clustering of unhealthy lifestyles, the proportion of at least two unhealthy lifestyles was higher among adolescents in the present study (93.6%) than Malaysian (56.6%) and Brazilian adolescents (85.5%) [25, 52] and Spanish adults (77.8%) [31]. The current status of each lifestyle will be discussed below.

A healthy diet encompasses a variety food groups such as fruits, vegetables, whole grains, starches, good fats and lean proteins [53]. This balanced diet not only helps prevent all types of malnutrition but also plays a crucial role in reducing the risk of NCDs including diabetes, heart disease, stroke and cancer [3]. However, in the present study, 86.4% of adolescents exhibited unhealthy dietary behaviors. Of particular concern was the inadequate consumption of vegetables, fruits and dried fruits, which was further exacerbated among adolescents resided in country, with low parental literacy and low family income. Vegetables and fruits were sources of many essential nutrients, and dried fruit consumption was associated with higher diet quality and lower weight or obesity indicators, and has beneficial effects on the prevention of metabolic syndrome [54–56]. We had known that socio-economic status (SES) in childhood (family income was an important component) had lasting effects on feeding management in adulthood [57]. The association between low SES and low vegetable and fruit consumption in adolescents also had been mentioned in previous studies, but the association between SES and dried fruit consumption has not been addressed [58]. Our findings supplemented the wider range of socio-demographic characteristics of adolescent fruit, vegetable and dried fruit intake and the associations besides SES.

Due to changes in lifestyle and dietary habits, the consumption of fast food has increased dramatically and most people do not eat breakfast every day, and the rate was higher than that in an Iranian study [59]. Our findings revealed a significant association between the consumption of fast food and breakfast habits and school type. Specially, university students emerged as the most frequent consumers of fast food, while also exhibiting the lowest frequency of breakfast consumption. This was supported by a previous study in which more than half of university students had lower quality diets and were charactered by low carbohydrate consumption (38.72% of total energy intake (TEI)) and high lipid intake (39.08% of TEI) [60, 61]. Notably, some studies have reported that children of highly educated parents were more likely to eat breakfasts regularly than children of less educated parents [62]. However, our study did not find associations between parental education and adolescents’ breakfast consumption, and some studies in China have produced similar results to ours [63, 64]. Adolescents with more educated parents consumed more fast food, which may be related to their parents’ employment status [65]. Parents with high educational levels tend to provide more social contributions at work and may not allocate enough time to prepare meals for their children [66]. In addition, we found that adolescents without close friends had the worst breakfast habits, but a previous study found no such link [67]. Throughout life, peers may influence adolescents positively or negatively. Eating breakfast with others could promote breakfast habits among adolescents, so making friends with peers who had healthy dietary habits would be beneficial for personal health [68]. These findings suggested that interventions to improve diet quality among adolescents were important, with particular attention to the effects of school type, family income, parental literacy and number of close friends on adolescents’ dietary behavior.

The present study found a positive correlation between alcohol and tobacco use which were potentially addictive behaviors that had serious negative effects on the academic performance and health status of adolescents [17]. Only 8.3% of Chinese adolescents smoked in 2003, while the percentage rose to 8.6% in 2018, and from 1990 to 2016 the rate of alcohol consumption rose from 15% to 18.2% [69]. In the current study, the overall prevalence of drinking and smoking behaviors among adolescents was low, but there were significant prevalence differences. Students who were in vocational high school, male, lived in country and with low family income were risk groups. Notably, we found that adolescents with light study burden had a high frequency of tobacco consumption. Lighter self-reported study burden did not indicate better academic performance, which may be due to the low academic focus among these adolescents [70]. The present study found that adolescents with 1–2 close friends were most likely to smoke and drink, consistent with previous studies [71, 72]. Peer drinking had been reported to influence adolescents’ alcohol expectations. Therefore, making friends with non-drinking peers should be encouraged to delay or reduce the frequency of alcohol use among adolescents [73].

Differences in parental literacy were not found to be significantly related in alcohol behavior, but adolescents with low parental literacy smoked more frequently, which may be partly explained that children were more likely to try smoking due to exposure to secondhand smoke, or poor family finances, stressful living conditions, and educational neglect [74]. The gradual decline in adolescents’ tobacco and alcohol use in many Western countries since the early twentieth century showed that regulating youth tobacco and alcohol behaviors under the policy environment was effective [75], and this had referential value for China.

Inadequate PA was a common problem in monitoring programs in different countries [13, 15]. It was already well known that men had a significantly better PA situation than women. It was noteworthy that university students with more discretionary time had the worst PA. Surveys indicated that their lack of PA may be due to lack of time (commitment to other activities not related to physical activity) and fatigue [76]. High parental literacy was negatively associated with PA of adolescents, and the explanation may be similar to its effect on diet. The lack of companionship of parents would be related to the decrease in children’s PA levels [66]. In this study, we found that only a minority of participants (around 30%) had healthy PA behaviors. This was similar to the pass rate obtained by Fan et al. in their 2016 study (29.9%) [77], which may indicate that the PA level of Chinese adolescents had not improved significantly in the past five years. In addition to improvements in physical health, appropriate PA has positive protective and enhancing effects on adolescents’ mental health and well-being [30]. Therefore, society and schools should take measures to improve the PA level of adolescents.

With the rapid socio-economic and internet development, children and adolescents were exposed to excessive ST. A previous study in China reported that about 56.3% of adolescents exhibited excessive ST [78]. The prevalence of high ST in this study was slightly lower than before, but showed significant groups differences. In addition to university students being a common group with high ST, ST was also relatively higher among adolescents living in country, with small families and being only child, similar to previous studies, and these factors may correspond to fewer family and social activities and greater tendencies for individuals to spend time on electronic devices [79]. However, excessive ST may displace time that should be spent on learning, physical activity and peer-related social activities, further affecting students’ academic performance, interpersonal relationships and physical health [65]. We did not find differences in ST in the number of close friends and study burden, and one possible explanation was that the number of close friends and study burden had complex effects on individuals, including possibly positive and negative factors.

Sleep deprivation could lead to drowsiness, fatigue, mood swings, irritability and reduced alertness, as well as an increased risk of stroke [21]. Due to the unique nature of the Chinese education system, most adolescents in school struggle to get enough sleep. Around 60% of the adolescents in this study were sleep deprived, which was much higher than the figure reported in the US (40.3%), and a previous study in China found that 26.9% of adolescents suffered from insomnia, with a high prevalence in high school students (31.8%) [80]. These had similar results to our study. In addition, the prevalence of sleep deprivation was also higher among adolescents living in city and with high family incomes, possibly due to the increased standard of living, the higher expectations of parents and the increased pressure on adolescents to study as a means of compressing sleep to get better academic results [9].

## Strengths and limitations

Our study has several strengths. Firstly, the sample size of this study was large, covering all types of schools in both city and country areas, which increase the significance of the results, so that the final sample can be considered to be more representative. Secondly, a detailed questionnaire was administered to a wide range of classical lifestyles and then clusters were used to more accurately assess the characteristics of the population at risk. Thirdly, the data of unhealthy lifestyles were relevant to socio-demographics and could be used to facilitate subsequent targeted interventions.

In the meantime, this study also had some limitations. Firstly, the research design of the cross-sectional study may have led to some information bias and inability to determine causality or direction. Therefore, it was essential to conduct cohort studies on lifestyle surveys of adolescents. Secondly, socio-demographic characteristics and lifestyle responses were self-reported and retrospective, potentially containing recall bias. To minimize this impact, questionnaires were on-site checked by professional staff during the survey. Thirdly, the non-standardized indicators used to define unhealthy lifestyles in this study hindered comparisons with similar studies and introduced result heterogeneity. To address this, we aligned our criteria with established standards from previous studies. Finally, the present study was conducted in Zhengzhou city, China, which may affect the extrapolation of the findings to some extent. Notably, the population of Zhengzhou city (one of representative cities in central China) was more than 12 million, and the gross domestic product (GDP) in RMB was 126.91 billion in 2021 and increased to 129.34 billion in 2022 [81, 82].

## Conclusions

Unhealthy lifestyles such as unhealthy dietary habits, inadequate PA, sleep deprivation and excessive ST remain prevalent among adolescents in China. These unhealthy lifestyles tend to be clustered and closely related to socio-demographic characteristics. Families and schools need to work together to improve the lifestyles of adolescents and to more effectively prevent future NCDs. China has set the goal of “Health China 2030”, which will further strengthen health education for adolescents. Further investigations may include long-term follow-up studies on adolescent behavioral trends and the development of targeted multi-behavioral intervention programs for high-risk groups.

## Supplementary Information

**Additional file 1. **Figure legends.

**Additional file 2. **Figure S1.

**Additional file 3. **Figure S2.

**Additional file 4. **Table S1.

**Additional file 5. **Table S2.

## Data Availability

The datasets used and/or analyzed during the current study are available from the corresponding author on reasonable request.
