# Supplementary material for: Unhealthy lifestyles and clusters status among 3637 adolescents aged 11–23 years: a school-based cross-sectional study in China
Source: BMC Public Health. 2023 Jul 3;23:1279. doi: 10.1186/s12889-023-16197-3 (PMC10318770; doi:10.1186/s12889-023-16197-3)
Supplement: Supplementary file 1 — Additional file 1. Figure legends. [file 12889_2023_16197_MOESM1_ESM.docx]

**Figure legends for supplemented figures S1-S2**

**Fig.S1 Study participant flow diagram**

Note: Due to graduation practice, senior three students in vocational high school in the city area of Zhengzhou did not participate in the survey, so we investigated 4351 students in total, and 3637 questionnaires were included in the analysis.

**Fig.S2 Analysis of the relevance of the six lifestyle categories**

The Spearman correlation analysis was used to explore the links between six lifestyle categories. Note: *P<0.05, **P<0.01.

Unhealthy dietary behavior: Diet quality score of 4 and above; Unhealthy alcohol behavior: Drank at least one glass of wine in the last month; Unhealthy tobacco behavior: Smoked at least one day in the last month; Unhealthy PA behavior: PARS-3 scores between 0 and 19; Unhealthy ST behavior: Daily average ST > 2 hours in the last month; Unhealthy SD behavior: Sleep deprivation in the last week (less than 9h/d for middle school students, less than 8h/d for high school and vocational high school students, less than 7h/d for university students).

**Abbreviations:** PA, physical activity; PARS-3, physical activity rating scale-3; ST, screen time; SD, sleep duration.
