# Supplementary figures and images for: Unhealthy lifestyles and clusters status among 3637 adolescents aged 11–23 years: a school-based cross-sectional study in China
Source: BMC Public Health. 2023 Jul 3;23:1279. doi: 10.1186/s12889-023-16197-3 (PMC10318770; doi:10.1186/s12889-023-16197-3)

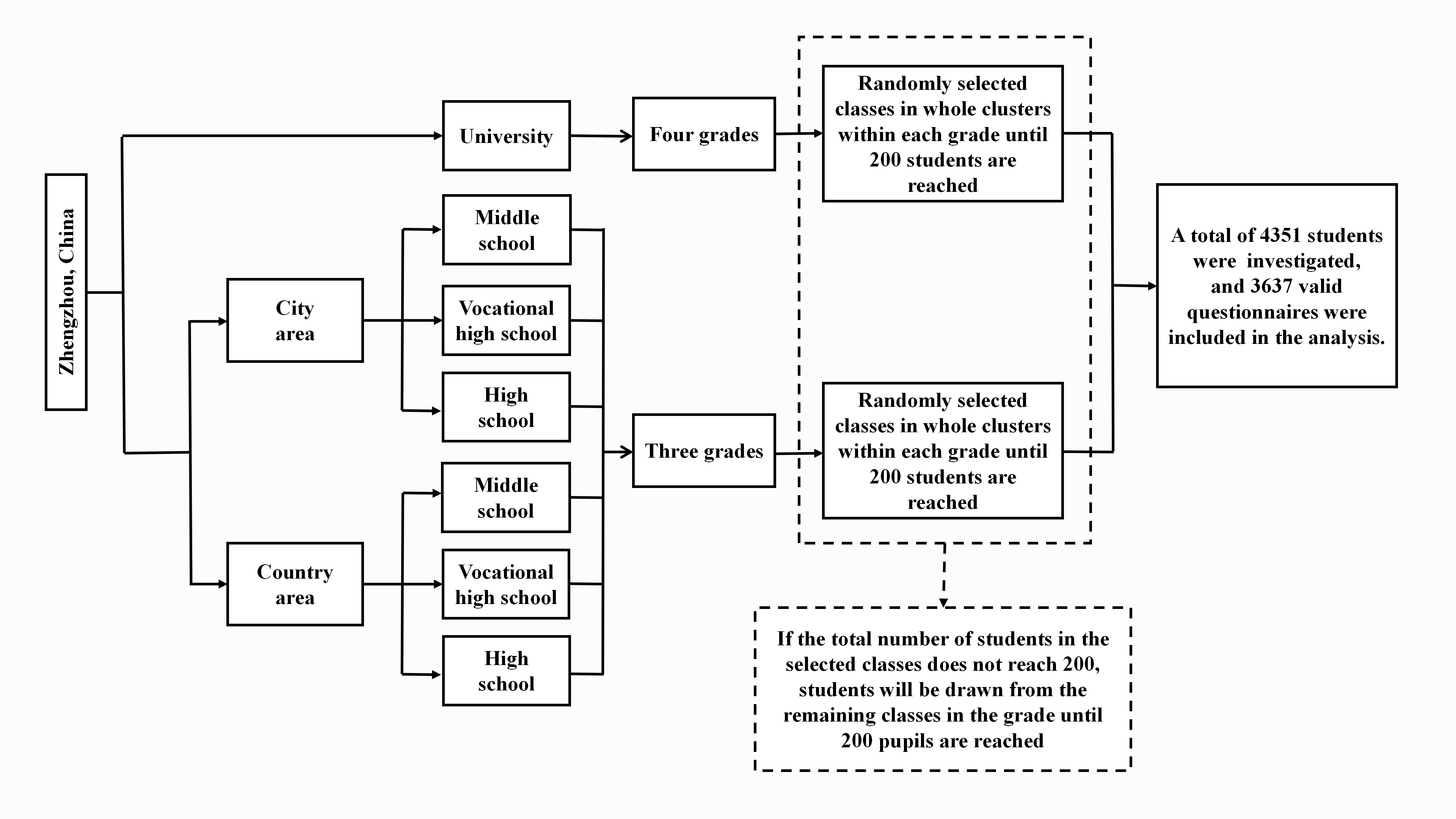

Supplement: Supplementary file 2 — Additional file 2. Figure S1. [file 12889_2023_16197_MOESM2_ESM.tif]

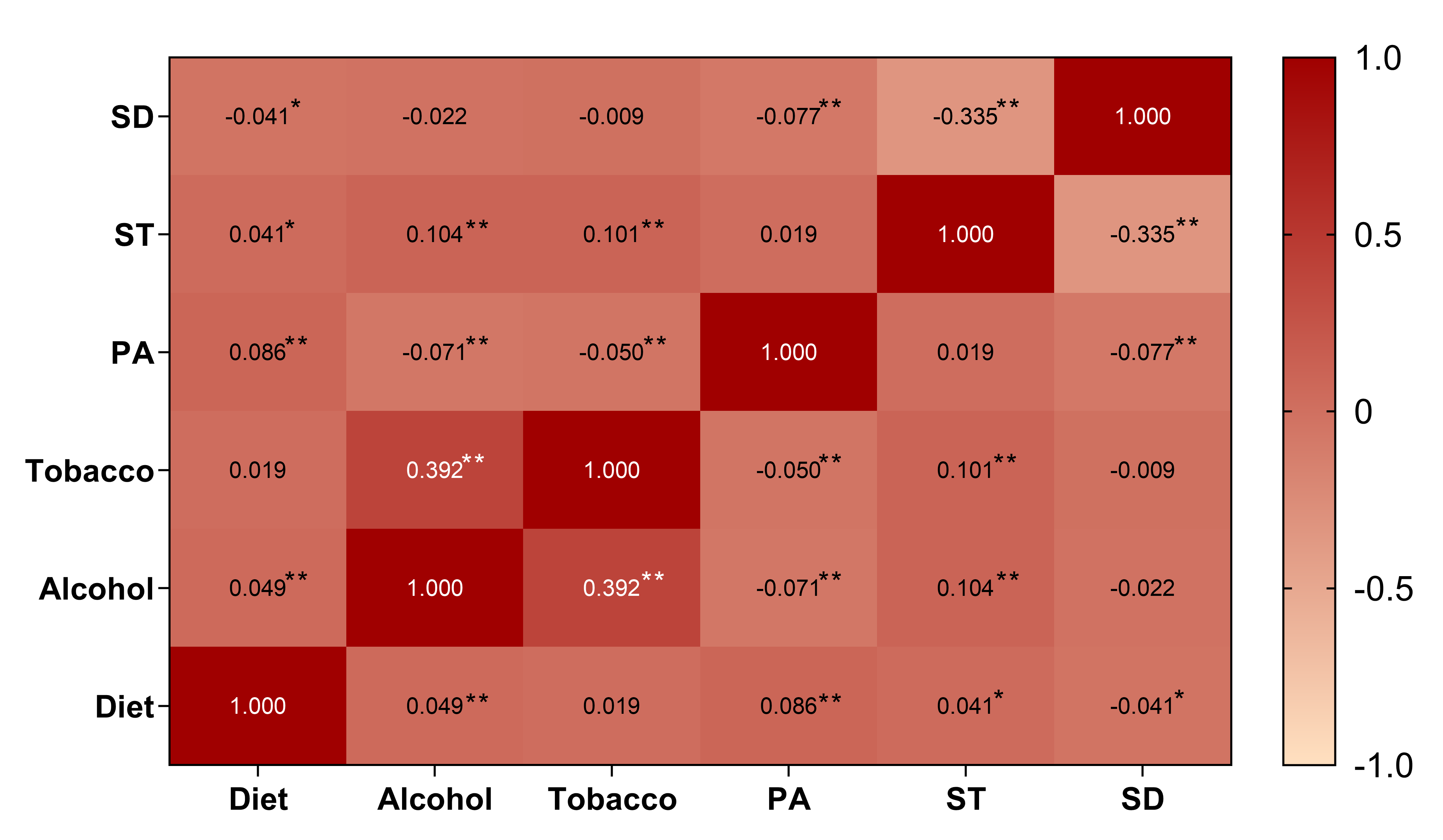

Supplement: Supplementary file 3 — Additional file 3. Figure S2. [file 12889_2023_16197_MOESM3_ESM.tif]
