# Supplementary material for: Unhealthy lifestyles and clusters status among 3637 adolescents aged 11–23 years: a school-based cross-sectional study in China
Source: BMC Public Health. 2023 Jul 3;23:1279. doi: 10.1186/s12889-023-16197-3 (PMC10318770; doi:10.1186/s12889-023-16197-3)
Supplement: Supplementary file 4 — Additional file 4. Table S1. [file 12889_2023_16197_MOESM4_ESM.docx]

**Table S1. Six categories of lifestyles among participants (N=3637)**

| **Variables** |  | **Diet** | |  | **Alcohol** | |  | **Tobacco** | |  | **Physical activity** | |  | **Screen time** | |  | **Sleep duration** | |
| --- | --- | --- | --- | --- | --- | --- | --- | --- | --- | --- | --- | --- | --- | --- | --- | --- | --- | --- |
|  |  | **Healthy** | **Unhealthy** |  | **Healthy** | **Unhealthy** |  | **Healthy** | **Unhealthy** |  | **Healthy** | **Unhealthy** |  | **Healthy** | **Unhealthy** |  | **Healthy** | **Unhealthy** |
|  |  | **N (%)** | |  | **N (%)** | |  | **N (%)** | |  | **N (%)** | |  | **N (%)** | |  | **N (%)** | |
| **School type** |  |  |  |  |  |  |  |  |  |  |  |  |  |  |  |  |  |  |
| Middle school |  | 204(20.3) | 800(79.7) |  | 895(89.1) | 109(10.9) |  | 963(95.9) | 41(4.1) |  | 355(35.4) | 649(64.6) |  | 669(66.6) | 335(33.4) |  | 132(13.1) | 872(86.9) |
| High school |  | 116(11.3) | 912(88.7) |  | 894(87.0) | 134(13.0) |  | 990(96.3) | 38(3.7) |  | 276(26.8) | 752(73.2) |  | 915(89.0) | 113(11.1) |  | 58(5.6) | 970(94.4) |
| Vocational high school |  | 91(10.8) | 754(89.2) |  | 663(78.5) | 182(21.5) |  | 725(85.8) | 120(14.2) |  | 227(26.9) | 618(73.1) |  | 443(52.4) | 402(47.6) |  | 512(60.6) | 333(39.4) |
| University |  | 83(10.9) | 677(89.1) |  | 658(86.6) | 102(13.4) |  | 740(97.4) | 20(2.6) |  | 152(20.0) | 608(80.0) |  | 71(9.3) | 689(90.7) |  | 611(80.4) | 149(19.6) |
| χ² |  | 53.724** |  |  | 47.036** |  |  | 31.824** |  |  | 52.481** |  |  | 183.355** |  |  | 1508.828** |  |
| **Gender** |  |  |  |  |  |  |  |  |  |  |  |  |  |  |  |  |  |  |
| Male |  | 266(14.9) | 1517(85.1) |  | 1399(78.5) | 384(21.5) |  | 1595(89.5) | 188(10.5) |  | 727(40.8) | 1056(59.2) |  | 1010(56.6) | 773(43.4) |  | 581(32.6) | 1202(67.4) |
| Female |  | 228(12.3) | 1626(87.7) |  | 1711(92.3) | 143(7.7) |  | 1823(98.3) | 31(1.7) |  | 283(15.3) | 1571(84.7) |  | 1088(58.7) | 766(41.3) |  | 732(39.5) | 1122(60.5) |
| χ² |  | 5.319* |  |  | 140.178** |  |  | 126.424** |  |  | 294.872** |  |  | 1.546 |  |  | 18.741** |  |
| **Residence** |  |  |  |  |  |  |  |  |  |  |  |  |  |  |  |  |  |  |
| Country |  | 240(11.2) | 1903(88.8) |  | 1817(84.8) | 326(15.2) |  | 1982(92.5) | 161(7.5) |  | 445(20.8) | 1698(79.2) |  | 1187(55.4) | 956(44.6) |  | 920(42.9) | 1223(57.1) |
| City |  | 254(7.0) | 1240(83.0) |  | 1293(86.5) | 201(13.5) |  | 1436(96.1) | 58(3.9) |  | 565(37.8) | 929(62.2) |  | 911(61.0) | 583(39.0) |  | 393(26.3) | 1101(73.7) |
| χ² |  | 25.247** |  |  | 2.197 |  |  | 20.505** |  |  | 127.62** |  |  | 11.26** |  |  | 105.476** |  |
| **Family population** |  |  |  |  |  |  |  |  |  |  |  |  |  |  |  |  |  |  |
| 1-3 |  | 70(15.6) | 379(84.4) |  | 377(84.0) | 72(16.0) |  | 430(95.8) | 19(4.2) |  | 129(28.7) | 320(71.3) |  | 199(44.3) | 250(55.7) |  | 181(40.3) | 268(59.7) |
| 4-5 |  | 318(13.7) | 2001(86.3) |  | 1985(85.6) | 334(14.4) |  | 2169(93.5) | 150(6.5) |  | 688(29.7) | 1631(70.3) |  | 1349(58.2) | 970(41.8) |  | 839(36.2) | 1480(63.8) |
| 6-15 |  | 106(12.2) | 763(87.8) |  | 748(86.1) | 121(13.9) |  | 819(94.2) | 50(5.8) |  | 193(22.2) | 676(77.8) |  | 550(63.3) | 319(36.7) |  | 293(33.7) | 576(66.3) |
| χ² |  | 2.995 |  |  | 1.105 |  |  | 3.47 |  |  | 17.767** |  |  | 44.267** |  |  | 5.598 |  |
| **Only child** |  |  |  |  |  |  |  |  |  |  |  |  |  |  |  |  |  |  |
| Yes |  | 77(17.3) | 367(82.7) |  | 379(85.4) | 65(14.6) |  | 427(96.2) | 17(3.8) |  | 149(33.6) | 295(66.4) |  | 198(44.6) | 246(55.4) |  | 178(40.1) | 266(59.9) |
| No |  | 417(13.1) | 2776(86.9) |  | 2731(85.5) | 462(14.5) |  | 2991(93.7) | 202(6.3) |  | 861(27.0) | 2332(73.0) |  | 1900(59.5) | 1293(40.5) |  | 1135(35.5) | 2058(64.5) |
| χ² |  | 6.091* |  |  | 0.009 |  |  | 4.297* |  |  | 8.448* |  |  | 35.503** |  |  | 3.488 |  |
| **Father’s educational level** |  |  |  |  |  |  |  |  |  |  |  |  |  |  |  |  |  |  |
| Primary school and below |  | 83(12.4) | 585(87.6) |  | 563(84.3) | 105(15.7) |  | 621(93.0) | 47(7.0) |  | 137(20.5) | 531(79.5) |  | 362(54.2) | 306(45.8) |  | 277(41.5) | 391(58.5) |
| Middle school |  | 207(12.5) | 1451(87.5) |  | 1416(85.4) | 242(14.6) |  | 1545(93.2) | 113(6.8) |  | 436(26.3) | 1222(73.7) |  | 1009(60.9) | 649(39.1) |  | 583(35.2) | 1075(64.8) |
| Senior high school and above |  | 204(15.6) | 1107(84.4) |  | 1131(86.3) | 180(13.7) |  | 1252(95.5) | 59(4.5) |  | 437(33.3) | 874(66.7) |  | 727(55.5) | 584(44.5) |  | 453(34.6) | 858(65.4) |
| χ² |  | 6.834* |  |  | 1.44 |  |  | 8.422* |  |  | 39.581** |  |  | 12.845* |  |  | 10.331* |  |
| **Mother’s educational level** |  |  |  |  |  |  |  |  |  |  |  |  |  |  |  |  |  |  |
| Primary school and below |  | 129(13.3) | 843(86.7) |  | 820(84.4) | 152(5.6) |  | 898(92.4) | 74(7.6) |  | 199(20.5) | 773(79.5) |  | 519(53.4) | 453(46.6) |  | 420(43.2) | 552(56.8) |
| Middle school |  | 175(11.8) | 1312(88.2) |  | 1287(86.6) | 200(13.4) |  | 1396(93.9) | 91(6.1) |  | 426(28.6) | 1061(71.4) |  | 927(62.3) | 560(37.7) |  | 489(32.9) | 998(67.1) |
| Senior high school and above |  | 190(16.1) | 988(83.9) |  | 1003(85.1) | 175(14.9) |  | 1124(95.4) | 54(4.6) |  | 385(32.7) | 793(67.3) |  | 652(55.3) | 526(44.7) |  | 404(34.3) | 774(65.7) |
| χ² |  | 10.756* |  |  | 2.459 |  |  | 8.678* |  |  | 40.545** |  |  | 23.166** |  |  | 29.626** |  |
| **Close friends** |  |  |  |  |  |  |  |  |  |  |  |  |  |  |  |  |  |  |
| 0 |  | 12(17.9) | 55(82.1) |  | 56(83.6) | 11(16.4) |  | 61(91.0) | 6(9.0) |  | 18(26.9) | 49(73.1) |  | 39(58.2) | 28(41.8) |  | 13(19.4) | 54(80.6) |
| 1-2 |  | 116(11.6) | 880(88.4) |  | 876(88.0) | 120(12.0) |  | 964(96.8) | 32(3.2) |  | 226(22.7) | 770(77.3) |  | 595(59.7) | 401(40.3) |  | 327(32.8) | 669(67.2) |
| 3-5 |  | 211(12.1) | 1529(87.9) |  | 1509(86.7) | 231(13.3) |  | 1642(94.4) | 98(5.6) |  | 448(25.7) | 1292(74.3) |  | 979(56.3) | 761(43.7) |  | 654(37.6) | 1086(62.4) |
| >6 |  | 155(18.6) | 679(81.4) |  | 669(80.2) | 165(19.8) |  | 751(90.0) | 83(10.0) |  | 318(38.1) | 516(61.9) |  | 485(58.2) | 349(41.8) |  | 319(38.2) | 515(61.8) |
| χ² |  | 25.174** |  |  | 25.93** |  |  | 38.138** |  |  | 61.009** |  |  | 3.243 |  |  | 16.047* |  |
| **Self-reported family income** |  |  |  |  |  |  |  |  |  |  |  |  |  |  |  |  |  |  |
| Low |  | 84(12.5) | 589(87.5) |  | 551(81.9) | 122(18.1) |  | 624(92.7) | 49(7.3) |  | 161(23.9) | 512(76.1) |  | 287(42.6) | 386(57.4) |  | 313(46.5) | 360(53.5) |
| Moderate |  | 333(12.8) | 2266(87.2) |  | 2254(86.7) | 345(13.3) |  | 2444(94.0) | 155(6.0) |  | 702(27.0) | 1897(73.0) |  | 1580(60.8) | 1019(39.2) |  | 907(34.9) | 1692(65.1) |
| high |  | 77(21.1) | 288(78.9) |  | 305(83.6) | 60(16.4) |  | 350(95.9) | 15(4.1) |  | 147(40.3) | 218(59.7) |  | 231(63.3) | 134(36.7) |  | 93(25.5) | 272(74.5) |
| χ² |  | 19.562** |  |  | 1.406* |  |  | 4.259 |  |  | 34.165** |  |  | 77.344** |  |  | 51.08** |  |
| **Self-reported study burden** |  |  |  |  |  |  |  |  |  |  |  |  |  |  |  |  |  |  |
| Light |  | 41(22.0) | 145(78.0) |  | 135(72.6) | 51(27.4) |  | 155(83.3) | 31(16.7) |  | 77(41.4) | 109(58.6) |  | 96(51.6) | 90(48.4) |  | 72(38.7) | 114(61.3) |
| Moderate |  | 291(13.3) | 1896(86.7) |  | 1899(86.8) | 288(13.2) |  | 2060(94.2) | 127(5.8) |  | 580(26.5) | 1607(73.5) |  | 1246(57.0) | 941(43.0) |  | 840(38.4) | 1347(61.6) |
| Heavy |  | 162(12.8) | 1102(87.2) |  | 1076(85.1) | 188(14.9) |  | 1203(95.2) | 61(4.8) |  | 353(27.9) | 911(72.1) |  | 756(59.8) | 508(40.2) |  | 401(31.7) | 863(68.3) |
| χ² |  | 12.117* |  |  | 28.326** |  |  | 40.617** |  |  | 18.94** |  |  | 5.602 |  |  | 16.092** |  |

**P*<0.05, ***P*<0.00
