# Supplementary material for: Unhealthy lifestyles and clusters status among 3637 adolescents aged 11–23 years: a school-based cross-sectional study in China
Source: BMC Public Health. 2023 Jul 3;23:1279. doi: 10.1186/s12889-023-16197-3 (PMC10318770; doi:10.1186/s12889-023-16197-3)
Supplement: Supplementary file 5 — Additional file 5. Table S2. [file 12889_2023_16197_MOESM5_ESM.docx]

| **Variables** |  | **Diet** |  | **Alcohol** |  | **Tobacco** |  | **Physical activity** |  | **Screen time** |  | **Sleep duration** |
| --- | --- | --- | --- | --- | --- | --- | --- | --- | --- | --- | --- | --- |
|  |  | **ρ** |  | **ρ** |  | **ρ** |  | **ρ** |  | **ρ** |  | **ρ** |
| **Diet** |  | 1 |  |  |  |  |  |  |  |  |  |  |
| **Alcohol** |  | 0.049^**^ |  | 1 |  |  |  |  |  |  |  |  |
| **Tobacco** |  | 0.019 |  | 0.392^**^ |  | 1 |  |  |  |  |  |  |
| **Physical activity** |  | 0.086^**^ |  | -0.071^**^ |  | -0.050^**^ |  | 1 |  |  |  |  |
| **Screen time** |  | 0.041^*^ |  | 0.104^**^ |  | 0.101^**^ |  | 0.019 |  | 1 |  |  |
| **Sleep duration** |  | -0.041^*^ |  | -0.022 |  | -0.009 |  | -0.077^**^ |  | -0.335^**^ |  | 1 |

**Table.S2 Analysis of the relevance of the six lifestyle categories**

**P*<0.05, ***P*<0.01
